# Maintenance of Voluntary Self-regulation Learned through Real-Time fMRI Neurofeedback

**DOI:** 10.3389/fnhum.2017.00131

**Published:** 2017-03-23

**Authors:** Fabien Robineau, Djalel E. Meskaldji, Yury Koush, Sebastian W. Rieger, Christophe Mermoud, Stephan Morgenthaler, Dimitri Van De Ville, Patrik Vuilleumier, Frank Scharnowski

**Affiliations:** ^1^Laboratory for Behavioral Neurology and Imaging of Cognition, Department of Neuroscience, University Medical CenterGeneva, Switzerland; ^2^Geneva Neuroscience CenterGeneva, Switzerland; ^3^Department of Radiology and Medical Informatics, CIBM, University of GenevaGeneva, Switzerland; ^4^Institute of Bioengineering, Ecole Polytechnique Fédérale de LausanneLausanne, Switzerland; ^5^Institute of Mathematics, Ecole Polytechnique Fédérale de LausanneLausanne, Switzerland; ^6^Department of Radiology and Biomedical Imaging, Yale University, New HavenCT, USA; ^7^Swiss Center for Affective SciencesGeneva, Switzerland; ^8^Psychiatric University Hospital, University of Zurich, ZürichSwitzerland; ^9^Neuroscience Center Zurich, University of Zurich and Swiss Federal Institute of Technology Zurich, ZürichSwitzerland; ^10^Zurich Center for Integrative Human Physiology, University of Zurich, ZürichSwitzerland

**Keywords:** neurofeedback, real-time functional magnetic resonance imaging (fMRI), self-regulation, brain training, maintenance, visual attention, visual imagery

## Abstract

Neurofeedback based on real-time functional magnetic resonance imaging (fMRI) is an emerging technique that allows for learning voluntary control over brain activity. Such brain training has been shown to cause specific behavioral or cognitive enhancements, and even therapeutic effects in neurological and psychiatric patient populations. However, for clinical applications it is important to know if learned self-regulation can be maintained over longer periods of time and whether it transfers to situations without neurofeedback. Here, we present preliminary results from five healthy participants who successfully learned to control their visual cortex activity and who we re-scanned 6 and 14 months after the initial neurofeedback training to perform learned self-regulation. We found that participants achieved levels of self-regulation that were similar to those achieved at the end of the successful initial training, and this without further neurofeedback information. Our results demonstrate that learned self-regulation can be maintained over longer periods of time and causes lasting transfer effects. They thus support the notion that neurofeedback is a promising therapeutic approach whose effects can last far beyond the actual training period.

## Introduction

Real-time fMRI neurofeedback is an emerging technique that allows to learn voluntary control over spatially localized brain activity (Weiskopf et al., 2004; deCharms, 2007; Sulzer et al., 2013). It can be used to study causal brain-function relationships by investigating how learned self-regulation of brain activity affects perception or behavior. For example, several studies have shown that self-regulation leads to behavioral effects that are specific to the functional role of the targeted cortical area (e.g., Weiskopf et al., 2003, 2004; deCharms et al., 2005; Bray et al., 2007; Caria et al., 2007; Rota et al., 2009; Shibata et al., 2011; Scharnowski et al., 2012). Real-time fMRI neurofeedback also holds great promises for clinical applications. For example, recent studies demonstrated therapeutic effects of neurofeedback training in chronic pain patients (deCharms et al., 2005), Parkinson’s disease (Subramanian et al., 2011), tinnitus (Haller et al., 2010), and depression (Linden et al., 2012).

However, in order for neurofeedback to be effective as a tool for cognitive enhancements or clinical applications, it needs to be shown that learned self-regulation transfers to situations where neurofeedback is not available anymore, and that learned self-regulation is maintained beyond the initial training period. Previous real-time fMRI neurofeedback studies already demonstrated that once learned, self-regulation can also be performed in transfer runs without feedback information, but this only for transfer runs immediately following the neurofeedback training (deCharms et al., 2004, 2005; Hamilton et al., 2011; Lee et al., 2012; Scharnowski et al., 2012; Sitaram et al., 2012; Ruiz et al., 2013). Unfortunately, published evidence of lasting neurofeedback training effects is very sparse. In healthy participants, two studies have shown that neurofeedback training induces plastic brain changes that last for at least 1 day after the training (Shibata et al., 2011; Harmelech et al., 2013). Another study found that neurofeedback-induced changes in resting state fMRI persisted for at least 2 months (Megumi et al., 2015). While this is by far the longest time period after training that has been investigated so far, the resting state effects demonstrate plastic changes, but they are not associated with applying learned self-regulation by the participants. In patients, only one study showed lasting changes due to neurofeedback training (Scheinost et al., 2013). Scheinost et al. (2013) found that patients with obsessive-compulsive disorder who successfully learnt to increase activity in the orbitofrontal cortex showed persistent changes in resting state connectivity and a significant reduction in contamination anxiety several days following the neurofeedback training. Even in the field of EEG neurofeedback, which has been used in research and in clinics for several decades and therefore has a much longer tradition than real-time fMRI-based neurofeedback, follow-up data has rarely been collected. The few studies which measured the effects of EEG neurofeedback over a period of up to 12 months to determine if self-regulation and its behavioral consequences are maintained, indicate that it remains stable (Tansey, 1993; Kotchoubey et al., 2001; Vernon et al., 2004; Leins et al., 2007; Kouijzer et al., 2008).

However, because EEG- and fMRI-based neurofeedback differ significantly with respect to the target areas and the physiological basis of the feedback signals, the findings obtained with EEG might not be transferable. We therefore re-scanned five participants 6 and 14 months after they successfully learned self-regulation of differential visual cortex activity, i.e., participants had learned to control the interhemispheric balance between their left and right visual cortex activity (Robineau et al., 2014). For the 6-month follow-up, participants were provided with the same neurofeedback information as for the initial training (top up session). For the 14-month follow-up, participants did not receive feedback information (transfer). We hypothesized that learned self-regulation is an acquired skill which can be maintained over longer periods of time. Specifically, we hypothesized that participants can still successfully self-regulate their visual cortex activity during the follow-up sessions, and that performance during the follow-up sessions is similar to that achieved at the end of the successful initial training period.

## Materials and Methods

**FIGURE 1:**
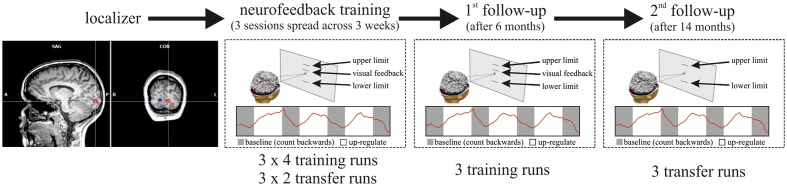
**Overview of the experimental Procedure.** In the first scanning session, the ROIs in the left and right visual cortex were defined with functional localizer runs. In three subsequent neurofeedback training sessions (on three different days spread across 3 weeks), participants then learned self-regulation of their visual cortex activity. Per training session, participants performed four training runs (of 2.8 min each) interleaved with two transfer runs (same as training runs but without feedback), i.e., the sequence of runs in each session was: training–training–transfer–training–training–transfer. Each training/transfer run was composed of four 20 s resting blocks (in gray) interleaved with three 30 s regulation blocks (in white). Six months after neurofeedback training participants performed another three training runs (first follow-up), and 14 months after the initial training participants performed three transfer runs without neurofeedback (second follow-up).

Details of the neurofeedback training study have been described previously (Robineau et al., 2014). In brief, participants were trained to control the differential feedback between a target region of interest in early visual cortex (*ROI*_target_), and its homolog in the opposite hemisphere (*ROI*_opposite_) (**Figure 1**). The ROIs were delineated in separate functional localizer scans and represented specific locations in the left and right visual fields. For the localizer, participants had to maintain fixation on a central point while a flickering checkerboard wedge (100% contrast, 8 Hz contrast reversal, 30° eccentricity along the horizontal meridian at a 45° angle) was presented on a gray background. The checkerboard was presented for three blocks of 30 s alternating in the left and in the right visual field, respectively, which were interleaved with baseline blocks during which participants fixated without any visual stimulation. Training participants to control the differential feedback signal was undertaken in three separate scanning sessions spread over the course of 3 days. For neurofeedback training, the activity difference between the two ROIs was fed back to the participant in the form of a visual thermometer display on a projection screen in the scanner bore. No other visual stimuli were presented. Neurofeedback training runs were interleaved with transfer runs during which participants performed learned self-regulation in the absence of feedback. For clarity, the main experimental parameters will be repeated here, but the methods section focuses on the follow-up extension of our previous study.

### MRI Data Acquisition

“All experiments were performed on a 3T MRI scanner (Trio Tim, Siemens Medical Solutions, Erlangen, Germany) at the Brain and Behavior Laboratory (University of Geneva). Functional images were obtained with a single-shot gradient-echo T2^∗^-weighted EPI sequence (30 slices, matrix size 64 × 64, voxel size = 4 mm × 4 mm × 4 mm, slice gap = 0.8 mm, flip angle α = 88°, bandwidth 1.56 kHz/pixel, TR = 2000 ms, TE = 30 ms), using a 12-channel phased array coil. The first three EPI volumes were automatically discarded to avoid T1 saturation effects. A T1-weighted structural image was acquired at the beginning of each scanning session (3D MPRAGE, 256 × 246 × 192 voxels, voxel size = 0.9 mm isotropic, flip angle α = 9°, TR = 1900 ms, TI = 900 ms, TE = 2.32 ms)” (Robineau et al., 2014).

Visual stimuli and instructions were displayed on a rectangular projection screen at the rear of the scanner bore, viewed through a mirror positioned on top of the head-coil. Stimulus display and response collection were controlled by MATLAB (MathWorks Inc., Natick, MA, USA) using the COGENT toolbox (developed by the Cogent 2000 team at the Wellcome Trust Centre for Neuroimaging and the UCL Institute of Cognitive Neuroscience, and Cogent Graphics developed by John Romaya at the Wellcome Department of Imaging Neuroscience).

The neurofeedback setup used Turbo-BrainVoyager 3.0 (Brain Innovation, Maastricht, The Netherlands), and custom scripts running on MATLAB. This allowed participants to be shown visual representations of BOLD signal changes in specific brain regions (in the form of a thermometer display projected into the scanner) with a delay of less than 2 s from the acquisition of the image. Head motion was corrected for in real-time using Turbo-BrainVoyager 3.0.

### Participants

In the initial study (Robineau et al., 2014), 14 participants took part, of which eight successfully learned control of differential visual cortex activity. We contacted all eight learners from the initial study and asked them to take part in a follow-up experiment. Only five were able to participate in the follow-up (five females, ages between 25 and 40 years, four right handed; see **Table 1** for details); the others did not respond or had moved away. All participants had normal or corrected-to normal vision. This study was carried out in accordance with the recommendations of the ethics committee of the University Hospital Geneva with written informed consent from all subjects. All subjects gave written informed consent in accordance with the Declaration of Helsinki. The protocol was approved by the ethics committee of the University Hospital Geneva. Before the follow-up scans, they received written instructions explaining that they will perform the same self-regulation task, which they had previously learned. The instructions included reminders about the neurofeedback thermometer display, and the cognitive strategy that they developed during the initial training period in order to successfully control the feedback signal (i.e., covert shifts of attention and imagery of moving visual stimuli). Furthermore, as for the initial training experiment, they were instructed to fixate on the central fixation point, to breathe steadily, and to remain as still as possible.

**Table 1:** Details about the participants who took part in the follow-up sessions.

Participant	Age	Gender	*ROI*_target_ hemisphere	Size of *ROI*_target_ (voxels)	Size of *ROI*_opposite_ (voxels)	First follow-up: top up session (months from initial training)	Second follow-up: transfer session (months from initial training)
1	27	Female	Right	45	32	7	14
2	27	Female	Left	37	30	–	14
3	40	Female	Right	38	24	7	14
4	27	Female	Right	15	22	6	13
5	25	Female	Right	18	9	3	9


### Follow-up Scanning Protocol

Participants took part in two follow-up scans. The first follow-up scan took place 3–7 months, and the second 9–14 months after the initial neurofeedback training sessions. Each follow-up scanning session started with a 5-min T1-weighted structural scan of the whole brain. This anatomical image was used for coregistration of the current head position with the T1-weighted structural scan obtained during the initial neurofeedback training using Turbo-BrainVoyager 3.0. The resulting coregistration matrix was used to transform the position of the ROIs used during the initial neurofeedback training into the correct location with respect to the current head position of the current follow-up session. This ensured that the same ROIs that were initially trained were also targeted in the follow-up sessions, although they took place months later. The visual ROIs had been determined by functional localizers in a separate scanning session before the initial neurofeedback training, (see, Robineau et al., 2014, for details).

After the structural scan, participants performed three neurofeedback training runs during the first follow-up session. As feedback signal, participants received the activity difference between the visual ROIs (left-right or right–left, depending on which configuration they were initially trained on). This activity difference was presented via a thermometer display. One participant did not perform the first follow-up scan (**Table 1**, participant 2). During the second follow-up scan participants performed three transfer runs. The transfer runs were identical to the training runs except that no neurofeedback information was provided to the participants, i.e., the thermometer reading was not visible.

The training and transfer runs were “composed of four 20 s baseline blocks interleaved with three up-regulation blocks of 30 s each (**Figure 1**). During the baseline blocks, the fixation cross at the center of the screen was black, which instructed the participant to mentally count backward from 100 in steps of -3 in order to maintain a stable baseline activity. During the up-regulation blocks, the fixation cross was white, which instructed the participant to now regulate their brain activity and increase the feedback signal. The background color of the screen was set to gray. The thermometer display consisted of a thin horizontal black line that moved up or down depending on the level of the differential feedback signal between the two visual ROIs (**Figure 1**). The differential feedback signal was presented throughout the training run (i.e., also during baseline blocks) and was updated every 2 s (i.e., once every TR). It was computed as the difference between the percentages of signal changes of the two visual ROIs (Eq. 1)” (Robineau et al., 2014):

$$f=100*\frac{\left({ROI}_{t\,\arg et}(up)-{ROI}_{t\,\arg et}\left(base\right)\right)}{{ROI}_{t\,\arg et}\left(base\right)}-100*\frac{\left({ROI}_{opposite}(up)-{ROI}_{opposite}\left(base\right)\right)}{{ROI}_{opposite}\left(base\right)}$$

where *f* is the current feedback signal, *ROI*_target_(*up*) is the average activity in the first ROI during up-regulation blocks, *ROI*_target_(*base*) is the average activity in the first ROI during baseline-regulation blocks, and *ROI*_contralateral_ is the same for the second ROI. For some participants, *ROI*_target_ was the left visual ROI, for others it was the right visual ROI (randomly assigned).

To avoid brisk fluctuations of the thermometer display, we applied temporal filtering by means of a sliding-window average over the previous three time points.

To normalize the percentage signal change values to the thermometer scale (which ranged from 25 steps below the fixation point to 25 steps above the fixation point; 5 pixels per step), the differential feedback signal values were scaled according to Eq. 2:

$$t_{m}=\frac{{psc}_{m}-{\lim it}_{low}}{{\lim it}_{up}-{\lim it}_{low}}*({Step}_{\max}-{Step}_{\min})+{Step}_{\min}$$

where *m* is the current time point, *t* is the temperature reading of the thermometer, *psc* is the percentage of signal change, *limit*_low_/*limit*_up_ are the mean of the five lowest/highest signal change values that have been acquired cumulatively up until the current time point, *Step*_max_ is 25 and *Step*_min_ is -25. The maximum and the minimum level of differential activity were indicated by thin black lines.

During the initial training study, we asked participants to perform a visual detection task and a line bisection task. These behavioral measures were not acquired in this follow-up study.

### Data Analysis

#### Functional MRI Preprocessing

“Offline data analysis used SPM8 (Wellcome Trust Centre for Neuroimaging, Queen Square, London, UK^1^) and BrainVoyager QX 2.6 (Brain Innovation). The images were corrected for slice time acquisition differences, realigned to the first scan of each run, and smoothed with an isotropic Gaussian kernel with 8 mm full-width-at-half-maximum (FWHM)” (Robineau et al., 2014).

#### Offline ROI Analysis

“The fMRI signal time-courses from the neurofeedback training and transfer runs were extracted from each visual ROI, averaged across voxels, demeaned, and detrended with linear and quadratic terms. Next, we specified GLMs with regressors for the up-regulation and the baseline conditions. The regressors were modeled as boxcar functions convolved with the canonical hemodynamic response function (HRF) in SPM8. The beta values for each regressor were fitted for the differential feedback signal for each run” (Robineau et al., 2014).

### Statistical Analysis

Our statistical analyses were conducted to test whether (a) the learning effect that we found in our initial study (Robineau et al., 2014) was also evident in the subgroup of the five participants that took part in the follow-up scans, and (b) they maintained performance during the follow-up scans. For these purposes, we performed an analysis of variance (ANOVA) based on the performance differences between the sessions (difference contrast) with the factor contrasts between sessions (three levels) and subject (to account for inter-subject variability) for both training/transfer data, respectively. Usually, absolute values of regulation performance are used in neurofeedback studies to assess learning across sessions. However, in the current study, we hypothesized that performance during the follow-up sessions is similar to that achieved during the initial training. To address this question, we considered performance differences between the sessions. To better estimate the mean performance and variance of the initial training, this ANOVA included the data of all learners for these time points (i.e., the estimates for sessions 1–3 include the data of the participants from the follow-up study and also the data from the three learners who were not able to take part in the follow-up study). In many longitudinal studies, observations are missing for some individuals. In that case, longitudinal differences can be tested using paired *t*-tests by keeping only individuals with complete records. Assuming that the underlying variance of the random error variable is homogeneous, this is not the optimal way to exploit the complete information contained in the data. Rather than estimating the variance for the comparison using only the data from the follow-up individuals, we can use the entire data to estimate it. This can be achieved by using an ANOVA and an assumption of homogeneity of the random error. The result of this ANOVA is a more stable estimate of the variance and an increase of the degrees of freedom in the reference Student-*t* distribution. This is a standard statistical procedure of borrowing strength across samples (Tukey, 1988; Dey and Rao, 2005; Hoaglin et al., 2011). Additionally, we performed a one-sample *t*-test to show that also for the subgroup of participants that performed the follow-up scans, the beta values are significantly positive in the last session of the initial training period. These analyses were calculated separately for the training and the transfer runs. All statistical tests were performed with R^2^.

## Results

**FIGURE 2:**
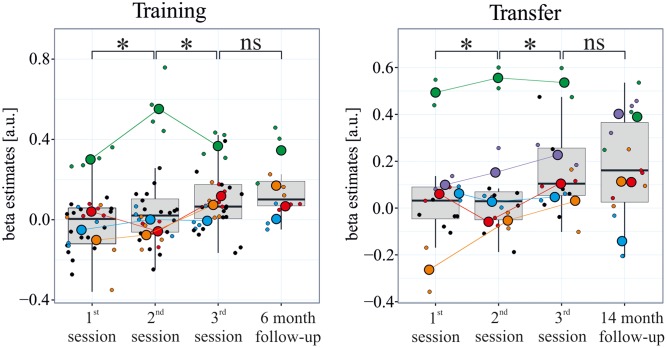
**Maintenance of learned self-regulation.** During the three initial neurofeedback training sessions which were spread across 3 weeks, participants learned to self-regulate their visual cortex activity. During a follow-up session with neurofeedback after 6 months, participants controlled the feedback signal as well as at the end of training. Even after 14 months, self-regulation was maintained, and this during transfer runs without neurofeedback. The rectangle represents the first and third quartiles; the band inside the rectangle represents the median; the whiskers extend from the rectangle to the highest/lowest value that is within 1.5 ^∗^ the inner-quartile range; small/large colored points represent individual runs/sessions of each participant of the follow-up study, respectively; black dots indicate runs of the learners who did not participate in the follow-up study (participants performed four training runs and two transfer runs per session during the original training, and three training and three transfer runs in the follow-up scanning sessions).

The subgroup of participants in the follow-up sessions successfully learned to control the differential feedback signal (**Figure 2**). The beta estimates increased significantly between sessions 1 and 2, and between sessions 2 and 3 [training: *F*(10,97) = 15.81, all *p*s < 0.01; transfer: *F*(10,52) = 12.88, all *p*s < 0.01]. Also, the *t*-test showed significantly positive beta values in the last session of the initial training (training: *t* = 3.60, df = 31, *p* = < 0.01; transfer: *t* = 3.46, df = 15, *p* ≤ 0.01), indicating a reliable modulation of brain activity during regulation compared to baseline blocks. Importantly, performance during the follow-up sessions did not differ from performance in the last training/transfer session of the initial training study [training sessions: *F*(10,97) = 15.81, *p* = 0.21; transfer sessions: *F*(10,52) = 12.88, *p* = 0.29].

## Discussion

Our study showed for the first time that learned voluntary control over brain activity can be maintained for a substantial period of time. In follow-up sessions we found that participants were able to perform self-regulation with neurofeedback just as well as they did at the end of the initial training period which had taken place 6 months earlier. Moreover, 14 months after the initial training participants were still able to exert learned control, and this during transfer runs without further neurofeedback information. These preliminary results in a small sample indicate that neurofeedback training effects last, and that participants can use their learned skill far beyond the initial training.

Neurofeedback based on real-time fMRI neurofeedback is a rapidly growing field because it allows for learning voluntary control over localized brain activity. To date, several studies show that neurofeedback training causes behavioral effects that are specific to the functional role of the targeted cortical area (Weiskopf et al., 2004; Bray et al., 2007; Caria et al., 2007; Rota et al., 2009; Shibata et al., 2011; Scharnowski et al., 2012, 2015; Robineau et al., 2014; Koush et al., 2015; Scharnowski and Weiskopf, 2015). Even more importantly, real-time fMRI neurofeedback training has also been shown to have therapeutic effects in chronic pain patients (deCharms et al., 2005; Guan et al., 2015), Parkinson’s disease (Subramanian et al., 2011), tinnitus (Haller et al., 2010), depression (Linden et al., 2012; Young et al., 2014), obsessive-compulsive disorder (Scheinost et al., 2013, 2014), spider phobia (Zilverstand et al., 2015), and addiction (Li et al., 2013; Karch et al., 2015; Kirsch et al., 2015; Hartwell et al., 2016). Especially for clinical applications of neurofeedback it is crucial that the learning effects persist beyond the initial training period and that voluntary control transfers to situations without neurofeedback information.

Our study significantly extends previous investigations which showed that neurofeedback training effects are still present 1 day (Shibata et al., 2011; Harmelech et al., 2013), several days (Scheinost et al., 2013), or 2 months after the training (Megumi et al., 2015). Firstly, we demonstrate that learned self-regulation is maintained for at least 14 months, which is by far the longest time period that has been investigated so far (Shibata et al., 2011; Harmelech et al., 2013; Scheinost et al., 2013; Megumi et al., 2015). One of the factors that probably contributed to such long lasting effects is that – like Scheinost et al. (2013) and Megumi et al. (2015) – we trained participants over several days, which gave them the chance to consolidate their learning overnight. Sleep has been shown to be beneficial for memory encoding and learning, which may have enhanced the persistence of learned self-regulation (Walker and Stickgold, 2004; Abel et al., 2013). Our results are in line with previous findings from the field of EEG-based neurofeedback, where follow-up studies revealed that learned self-regulation and its behavioral consequences are maintained for at least 12 months (Tansey, 1993; Kotchoubey et al., 2001; Vernon et al., 2004; Leins et al., 2007; Kouijzer et al., 2008).

Secondly, we show that self-regulation can be applied months after the initial training without neurofeedback information. That learned control over brain activity transfers to situations where neurofeedback is no longer available is another key requirement for clinical uses of neurofeedback (Stoeckel et al., 2014). Transfer effects have been previously demonstrated immediately following neurofeedback training (deCharms et al., 2004, 2005; Hamilton et al., 2011; Lee et al., 2012; Scharnowski et al., 2012; Sitaram et al., 2012; Ruiz et al., 2013), but here we extend these findings by demonstrating that transfer effects last for several months. To promote transfer, we interleaved the neurofeedback training with transfer runs without neurofeedback (Robineau et al., 2014), which might have contributed to the persisting transfer performance. Finally, the lasting changes that we found relate to self-regulation skills that can be applied voluntarily by the trained participants rather than to concomitant training effects such as the previously reported persistent resting state changes (Scheinost et al., 2013; Megumi et al., 2015).

## Limitations

The main limitation of this study is the small sample size of only four participants in the first follow-up session and five participants in the second follow-up session. Thus, our results should be considered as a preliminary demonstration of lasting effects that require further verification in larger samples. The second limitation is that we did not include a control group without neurofeedback that attempts self-regulation based only on cognitive task instructions. Without such a control group, we cannot completely exclude the possibility that mere practice led to the improvement across sessions during the initial training and transfer runs, and that these practice effects could have then carried over to the follow-up study. However, in the original training study, the non-learners also practiced self-regulation for as long as did the learners, but they did not show improvements. Moreover, other real-time fMRI neurofeedback studies that included control groups who received either sham feedback or no feedback have firmly established that neurofeedback is necessary for learning to self-regulate brain activity [e.g., for the anterior cingulate cortex (Hamilton et al., 2011), for the inferior frontal gyrus (Rota et al., 2009), and most importantly for the visual cortex (Shibata et al., 2011; Scharnowski et al., 2012)]. Finally, in this follow-up study, we did not ask participants to perform the visual detection and line bisection tasks that they performed during the original training. Hence, long-lasting behavioral consequences of neurofeedback training need to be addressed in future follow-up studies.

## Conclusion

Taken together, we show in a small sample of healthy participants that learned self-regulation of differential visual cortex activity can be maintained over long periods of time and transfers to situations without neurofeedback. These findings are likely not specific for learned control over visual cortex activity, but probably also apply to other neurofeedback target regions. The visual cortex is an area that has been shown to be less responsive to neurofeedback training (Harmelech et al., 2015), which is also reflected by the relatively large number of participants who failed to learn to control visual cortex activity (Scharnowski et al., 2012; Robineau et al., 2014). Moreover, compared to standard ROI-based feedback, the differential feedback signal that was used in the present study likely is more difficult to control because uncorrelated Gaussian noise of the signals from the two ROIs is additive and may thus reduce the signal-to-noise ratio (SNR). Hence, our results imply that neurofeedback training with real-time fMRI causes plastic brain changes rather than just short-lived state changes. Although the neural mechanisms underlying these lasting changes are still unknown (Sitaram et al., 2016), evidence for such lasting changes as well as for transfer to situations without neurofeedback support the potential of neurofeedback as a promising novel experimental therapy for neurological and psychiatric conditions.

## Author Contributions

FR, PV, and FS conceived and designed the experiments. FR performed the experiments. FR, DM, YK, SR, CM, SM, DV, and FS analyzed the data/contributed analysis tools. FR and FS wrote the paper and all authors critically reviewed and approved the final manuscript.

## Conflict of Interest Statement

The authors declare that the research was conducted in the absence of any commercial or financial relationships that could be construed as a potential conflict of interest.

## Footnotes

1 http://www.fil.ion.ucl.ac.uk/

2 https://cran.r-project.org
